# A Day in the Life: Secondary School Students’ Experiences of School-Based Physical Activity in Ireland, Finland, and the United States

**DOI:** 10.3390/ijerph19031214

**Published:** 2022-01-22

**Authors:** Jaimie McMullen, Collin Brooks, Cassandra Iannucci, Xiaoping Fan

**Affiliations:** 1 School of Sport and Exercise Science, University of Northern Colorado, Greeley, CO 80639, USA; 2 Department of Sport Management, Wellness, and Physical Education, University of West Georgia, Carrollton, GA 30118, USA; cbrooks@westga.edu; 3 Faculty of Arts and Education, Deakin University, Waurn Ponds, VIC 3216, Australia; cassandra.iannucci@deakin.edu.au; 4 Department of Educational Programs, Texas A&M International University, Laredo, TX 78041, USA; xiaoping.fan@tamiu.edu

**Keywords:** whole-school physical activity, student voice, comprehensive school physical activity program, school physical activity, high school

## Abstract

Internationally, there is an effort to have schools adopt a whole-school approach to physical activity promotion. Such a model includes physical activity opportunities throughout the whole school day, including physical education; before, during, and after school physical activity; and staff and community engagement. The purpose of this study was to describe the physical activity experiences of young people attending secondary schools in Finland, Ireland, and the United States where a whole-school approach to physical activity promotion was employed. One school in each country was identified based on its adoption of a national physical activity initiative (i.e., Finland—Finnish Schools on the Move; Ireland—Active School Flag; United States—Let’s Move Active Schools). Data were collected through observation with field notes, photos, and interviews with key stakeholders. The results are presented as analytic narrative vignettes that represent a “typical” school day. The results provide a glimpse into available physical activity opportunities for young people at each school and demonstrate an emphasis on active school culture.

## 1. Introduction

Around the world, most young people attend school for the majority of their waking hours. As a result, schools are well positioned to influence the physical activity behaviors of students by providing opportunities for them to be active while at school. Internationally, schools are being asked to provide physical activity opportunities in addition to those that already exist (i.e., physical education, club sports, etc.) through a whole-school approach [1]. Such a model includes opportunities to participate in physical activity before (e.g., active transportation), during (e.g., breaks, recess, and classroom movement), and after school (e.g., clubs, school sports), with staff engagement (e.g., employee wellness programs, staff leading physical activity for students) and parent/community involvement. In response, physical activity initiatives have been developed all over the world at local, regional, and national levels. For example, countries such as Ireland, Finland, Poland, and the United States have developed national initiatives to promote physical activity in schools [2]. These unique initiatives each have slightly different structures and components, but all seek to provide additional opportunities for young people to be active at school, and to ultimately achieve higher levels of physical activity.

In the United States, a whole-school approach is often discussed in the context of a comprehensive school physical activity program (CSPAP), which specifically includes all five components aligned with a multi-component approach (e.g., a combination of physical activity before and after school, physical education, physical activity during school, staff involvement, and family/community engagement) [3]. While much of the research on school-based physical activity promotion considers a single component (e.g., physical activity in the classroom), others attempt a more comprehensive, multi-component approach. For example, in a recent systematic review that considered 32 school-based physical activity studies, most included physical education plus one or two additional components (e.g., physical activity before or after school), while fewer (eight) included physical education and three additional whole-school components. Only one study included all five CSPAP components [4]. Despite the variability in the types of initiatives/interventions, it appears that multicomponent, whole-school approaches to physical activity promotion can result in positive outcomes for youth [4].

Conceptually, it is imperative to center the student as the consumer of school-based physical activity when considering the design and implementation of such programs. Students have been acknowledged as essential stakeholders in whole-school approaches to physical activity promotion [1]. Therefore, student voices should be amplified at all stages of development and implementation of whole-school physical activity initiatives, and their feedback should also be sought during the evaluation of such programs. From a socio-ecological perspective [5], the comprehensive school physical activity program (CSPAP) conceptual framework acknowledges various levels of influence that impact students’ access to physical activity opportunities at school [3]. However, despite this, it is unclear the extent to which young people are regularly consulted within whole-school physical activity promotion efforts. As a result, it seems important to consider the experiences of young people in order to better understand how and why they access these opportunities (and why they do not) in order to improve whole-school physical activity programs. Therefore, the purpose of this study was to describe the physical activity experiences of young people attending secondary schools in Finland, Ireland, and the United States where a whole-school approach to physical activity promotion was employed.

## 2. Materials and Methods

### 2.1. Context

One school in each country was identified based on its adoption of a national whole-school physical activity initiative (i.e., Finland—*Finnish Schools on the Move*; Ireland—*Active School Flag*; United States—*Let’s Move Active Schools*). In the United States, *Let’s Move Active Schools* (now *Active Schools*; https://www.activeschoolsus.org/ (accessed on 1 December 2021)) includes the promotion of physical education and physical activity with the goal of helping students participate in 60 min of daily physical activity. The *Active School Flag* in Ireland requires schools to implement environmental changes that enable them to achieve a physically educated and physically active school community (http://www.activeschoolflag.ie (accessed on 1 December 2021)). Finally, the *Finnish Schools on the Move* program in Finland allows schools to apply to be part of the established network if they have developed and implemented a plan to increase opportunities for children to have several opportunities to be active at school throughout the school day (https://www.liikkuvakoulu.fi/english (accessed on 1 December 2021)). In addition to their adoption of a whole-school physical activity initiative, each school was specifically selected based on an existing relationship between members of the research team and the school personnel. Table 1 includes additional information about each school.

### 2.2. Data Collection

Access to each school was negotiated with the school principal and all participants ultimately provided informed consent and/or assent to participate in the study. During data collection, the lead author had full access to the school building(s) and the grounds, allowing her to move freely and observe the school environment throughout the school day. Data were collected by observation with field notes, photos, and interviews with key stakeholders (i.e., students, teachers, principals, and parents/community members).

#### 2.2.1. Observation with Field Notes

The lead author spent three days at each school (Tuesday–Thursday), from approximately 30 min before school started until approximately 30 min after the last bell, within a two-month period (i.e., March–May). These weekdays were selected to maintain consistency across sites. Notes were taken about the physical environment (inside and outside), the school day schedule, what students were doing during the day (particularly during break times), and anything else that connected with students’ access to physical activity at school. All notes were recorded using an iPad and were taken in real time, with a total of 30 single-spaced typed pages of field notes recorded.

#### 2.2.2. Photos

During observations, photos (N = 149) were taken using an iPad of indoor and outdoor spaces/equipment that could be or were being used for physical activity. All photos were taken on the school campus with the exception of the school in Finland, where photos were taken at a nearby community park that was frequently used for school-related activities (e.g., physical education). Additionally, photos were taken of print material and other signage in the schools that either promoted physical activity or depicted a message related to being physically active.

#### 2.2.3. Interviews with Key Stakeholders

Several key stakeholders were interviewed at each school, including students (N = 26), classroom teachers (N = 15), parents/community members (N = 9), and the school principals (N = 3). Each interview lasted approximately 40–65 min. The interviews were audio recorded and used a semi-structured focus group format with the exception of the school principals, who were interviewed individually. Interview questions provided participants with the opportunity to describe their knowledge of and experience with physical activity opportunities at their school from their perspective.

### 2.3. Analysis

The results are presented as analytic narrative vignettes [6] that represent a “typical” school day based on data collected across three days at each school. Vignettes use a story-like structure to convey a focused description of events and behaviors that are representative of the case(s) being studied [7]—in this case, the physical activity experiences of young people at each school. Vignettes are written based on patterns and themes identified during analysis. Therefore, the vignettes presented in this study do not represent the experiences of one particular participant, but instead provide an overall portrait that considers data from multiple interview participants, observations, and photos from the same school. This methodology was selected in order to provide the reader with narrative examples of what young people may experience related to physical activity opportunities in school and allows the researcher to provide a more in-depth interpretive analysis than simply reporting themes [6].

Data were analyzed inductively and the data corpus from each site was considered separately. Each of the first three authors were responsible for the initial analysis for a specific site (i.e., first author, Finland; second author, United States; and third author, Ireland). Several steps were taken in the analysis process, starting with multiple reads of all transcripts and observation notes, and a review of all photographs. Next, common events and/or behaviors were noted that appeared as patterns in the data, which were then organized into a logical order or list. After these patterns were identified, they were verified to ensure that they were truly representative of the data. Then, these events and behaviors were used to draft a narrative vignette that would help tell a story representative of the data. Finally, each narrative vignette was verified by an independent reader (the fourth author), who had conducted an independent analysis of all data, and a peer-debriefing process was used to resolve any issues related to the data-based accuracy of the vignettes. The guidelines for writing narrative vignettes vary, and often stray from typical scholarly discourse; however, vignettes are firmly rooted in data [7]. While all data were considered, student responses in the interviews were prioritized for the purposes of developing the results.

### 2.4. Trustworthiness

Trustworthiness was established in several ways. First, the same data collection protocols were used at each school, which meant that the same data were being collected across contexts. Second, several data sources were collected and triangulated, which allowed the researcher to avoid issues related to lack of evidentiary support [6]. Third, the narrative vignettes were developed based on patterns that existed across all data sources within each school [8]. Lastly, the use of the independent reader and a peer debriefing process contributed to establishing trustworthiness [6].

## 3. Results

The following narrative vignettes represent various experiences that young people had with physical activity opportunities at their secondary school. Two vignettes are presented from each school/country, and while these two representations are by no means generalizable to the experiences of each young person at each school, they are intended to provide a glimpse into the available physical activity opportunities and how students engage with them.

### 3.1. Finland

#### 3.1.1. Linnéa—8th Grade

**Figure 1:**
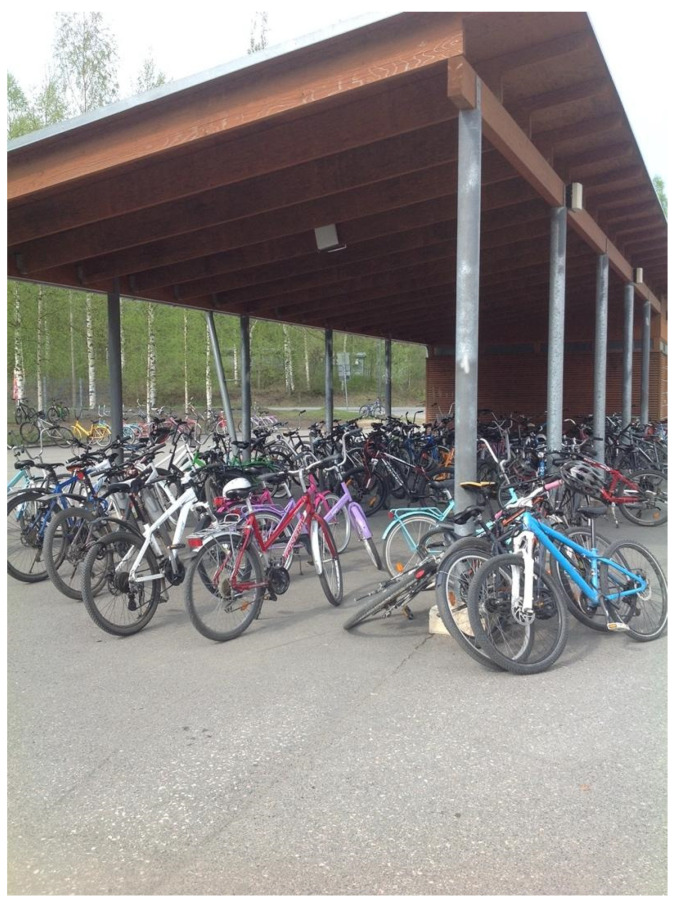
Bike storage.

One thing that I really like about school is that we don’t have to sit in the classroom all day. We get some breaks between classes that allows us to get up and go outside if we want. Well, during our exercise recess we are required to go outside, even when it is very cold—but during our other breaks we have time to sit on the benches in the hallway, or go outside. It’s kind of funny because we sit next to each other on the benches and we talk to each other through our phones—I sometimes wish they would just take away all the phones so that we would actually talk to each other more. If we are not sitting on the benches, my friends and I like to hang out on the swings or just walk and talk around the schoolyard. We do have one five-minute break and that just gives me enough time to check my phone and get my books for the next class. Honestly, one thing that annoys me is that boys have better PE classes than girls. It’s like they think girls are fragile and will break easily. There are times where I want to play more like we did in primary school, but I don’t always feel like it. A few years ago, some students got to help design the yard and we have some pretty cool things out there. There’s a spinning stool, a big blue swing, and other stuff—like there’s stuff for us to do, but like I said before, I’m not always in the mood. I always ride my bike to school (see Figure 1), its faster than getting dropped off in the car, but next year I will probably get a moped or a motorbike because a lot of the grade 9 students get to school that way. I do like riding my bike, but once I’m old enough I probably won’t do that anymore.

#### 3.1.2. Antti—7th Grade

**Figure 2:**
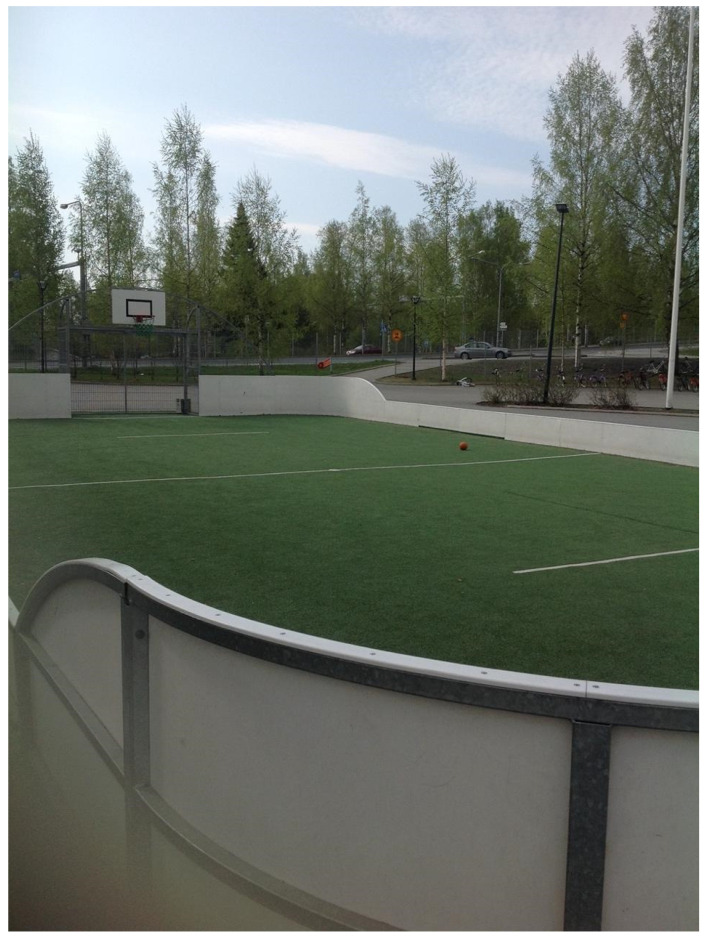
Outdoor mini-stadium.

During most breaks you can find me and my friends at the mini-stadium playing football or sometimes we play basketball on the sport court (see Figure 2). We like to compete against each other and will turn pretty much anything into a competition. Anyone can come play with us, but it is usually just the same group of us. I think I was more active at primary school than I am here. We have breaks between each class, one at the end of the day is just 5 min, but others are longer, like from 15 to almost 30 min, but I think there were more things to play and more people playing in primary school than at lower-secondary school. We get a lot of encouragement from the PE teacher and some other teachers who come out to make sure we are not being bad during breaks. It’s kind of cool when they jump in and take a shot or tell us that we are doing a good job. We are a “moving school” so I think that’s why we have all the breaks and the teachers compliment us for being active—but I don’t really know everything about it. Some of my friends play ping-pong during the breaks—those tables are all over the school. Like seriously, they are in every hallway and open space. I heard when they first got them there was always equipment to use to play, but now you have to go get the equipment, so that’s why I just come outside. It’s good that I use the breaks to play and be outside because unlike pretty much all of my friends, I don’t bike or walk to school. I live too far away, so I take the bus. If the bus gets to school early, then I can use that time to play football too—but usually I get here just in time to start my first class.

### 3.2. Ireland

#### 3.2.1. Robin—Transition Year

On a typical day I usually go to the morning drop-in training sessions in the gym before homeroom. Mr. Kavanagh supervises us doing some strength training. It’s mostly the rugby lads that drop in, but I find it good banter, so I’ll go along. The training session is open to everyone and isn’t structured around a program. Sometimes some of the girls drop in too. It’s nice to have the option otherwise I’d probably just meet up with my friends in the hallway until homeroom.

I play Gaelic football outside of school, I’m the captain of my team. Sometimes I wish we had a school Gaelic football team because I couldn’t be bothered playing hockey or rugby. My family is big into the GAA. There have been some students who have asked to start a team here, but our principal says we just don’t have the facilities; the pitch is booked solid between the school sports we have and community bookings. Our teachers and coaches are big into sport, they are supportive for all students to find something active they enjoy doing. But they specialize in hockey and rugby at this school; always winning titles, so they get the priority. I knew that coming here, so, fair enough. At least I can save my energy for proper training with my club in the evenings. And come exam time in 6th year I won’t need to choose between sport and study like the other lads.

When it’s not raining at lunch break they open the pitch for us to kick a ball around or play a game of touch rugby. There are definitely more options for us to stay active throughout the day when the weather is nice and we can go outside (besides dodging raindrops between classes when we need to change buildings). I find the pitch is locked up for most of winter, probably because it rains a lot. If we can’t kick around or go outside at lunch, I would usually meet my friends in the dining hall. I don’t stick around much after school. Most of my friends play on the school hockey team, so on the days they have training I just head home. I’m one of the few students who live close enough to walk to school.

#### 3.2.2. Grainne—Transition Year

I usually arrive at school before all my friends in the morning. It’s the only time my mammy can drop me off on her way into town for work. If it’s not raining, she lets me out at the roundabout, and I walk the rest of the way. It only takes about five minutes to walk, so I don’t mind. Nearly everyone who isn’t a boarder either gets a lift or a bus in so you can imagine how manic the car park gets in the morning! Come to think of it, I don’t know anyone who walks in. The majority of us live pretty far away, unless you’re a boarder in which case you’d live right on campus like my best friend Sinead. We play on the hockey team together and we have a really big match coming up with a chance to win the cup. We have such supportive coaches, like Miss O’Donnell, who always tells us she believes we will win. I’m not sure what sport I will do after the hockey season, but I am sure I will find something.

**Figure 3:**
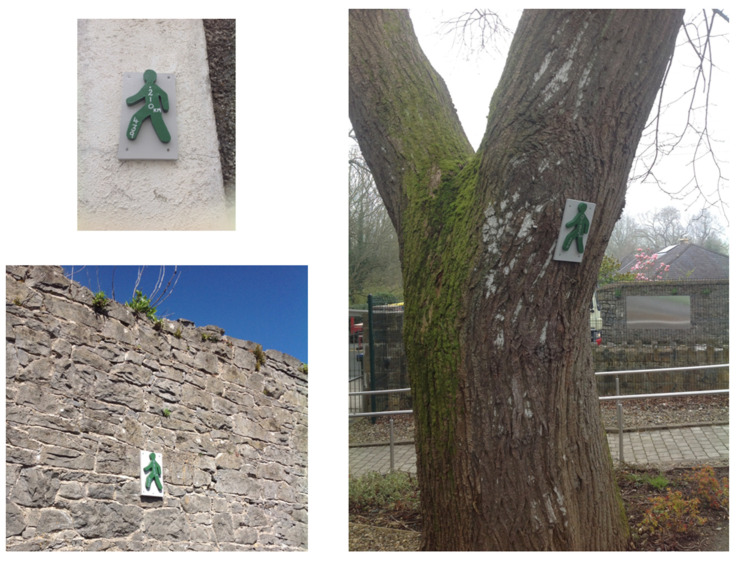
Walking path markers.

If we are at school early we usually walk around campus, there is a walking path sign-posted with “green men” that takes you clear around campus (see Figure 3). There is an elective PE class for the transition year students that is all about promoting physical activity in the school. They marked out this route for a school event walk we had earlier this year. Sometimes our PE teacher lets us walk along it if we don’t want to participate in class, but mostly we walk and talk around the path on dry days before school or during lunch. It gets a lot of use!

When we are walking, we often pass the boys kicking around a soccer ball. I sometimes imagine what it would be like to be able to just kick around a ball before homeroom. It would be nice, but me mam would give out to me for ripping my uniform lining! They certainly weren’t designed to withstand sport like that. Walking is alright though; between the walking path and walking between classes I get a lot of steps in throughout the day. It used to be that the teachers rotated around classrooms while we stayed in our homeroom. I’m glad that has changed. We don’t have a lot of time between classes, once the bell rings we are expected to go straight to our next class which could be in another building. There isn’t time to do much of anything between classes, but I’m grateful we aren’t sitting in the same desk all day.

### 3.3. United States

#### 3.3.1. Rilynn—11th Grade

**Figure 4:**
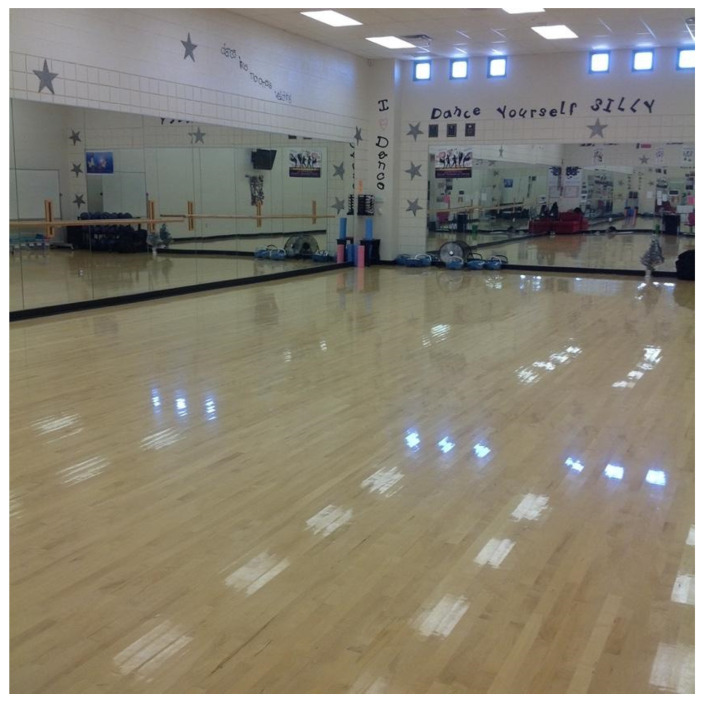
Dance studio.

On a typical day my mom drops me off at school on her way to work. She works downtown, so I can be here pretty early. I hate getting up so early, but at least it gives me time to catch up on homework. I usually sit outside the dance studio until my friends get here—and then I have dance first hour (see Figure 4). I’m not much into sports, so I love that we can take dance instead of PE. You only need one PE credit to graduate, but I’ve taken dance every semester since freshman year. What’s cool is that there is a school dance team and I’ve been on the intermediate team for the last two years, and this year I finally made advanced! I’m hoping I will make it again for next year.

Sports is like everything at this school, so if you don’t play the main sports there isn’t many other options to be active—except for dance. After dance, most of my day is spent sitting. We only get five minutes to get from one class to the next and in pretty much all of my classes we just sit. Our campus is big, so you need that whole five minutes to get to your next class. Every once in a while, one of our teachers will take us outside to do a lesson or an activity.

At lunch, after we eat, I sit around with my friends and we do stuff on our phones. I know my little brother goes to that lunchtime open gym thing that the local university students put on. I don’t know much about it, but I would never go there, don’t get me wrong, I think it’s a great idea, but I think it is mostly younger kids who go there. One day last week, my friends and I did walk the track at lunch because we are allowed to go out during our lunch to walk or play, but we don’t do that too often. Our lunch is long—like an hour—I kind of wish it wasn’t so long and we could start school later in the day.

Once the school day is over I get a ride home with one of my friends who is also on the dance team—both our parents work and since we sometimes have practice after school it works out great. I live pretty close to the school, but like no one I know bikes or walks to school, everyone takes the bus, drives themselves or gets rides.

#### 3.3.2. Aaron—12th Grade

Sports are kinda my life. I consider myself a leader on the football team and I start every game for the varsity team. I think that there are chances for all students to be part of some school sports because they are zero drop, which means everyone makes the team. My day starts early, for zero hour which is a weight lifting class just for athletes before the actual school day starts. We lift weights for football all year round—even in the summer. My parents and sister think I’m crazy for getting up so early, but our coaches require us to be there, so I don’t really have a choice. My sister does barrel racing rodeo stuff outside of school, so she is busy too but at least gets to sleep in. My first class of the day is English, and the teacher usually makes us sit for a long time. Every once in a while, we will be able to move around in class, but it is mostly sitting and listening. My other morning classes are pretty much the same. We sit for a long time at our desks.

At lunch my friends and I pretty much just chill. We have a whole hour for lunch, which is honestly too much time. I’ve heard announcement and I have seen a few signs around the halls that say they are doing sports at lunch in the gym. I think that most of the kids that go there are freshmen and are probably not athletes. After lunch, I have another class, and then we have a football class in seventh hour. I don’t have to do like an actual PE class because we have weights in the morning and football class in the afternoon. I have some friends that are in PE, and they say they really like it. I kind of wish I could do regular PE because they do activities like tennis and softball that sound super fun.

Coach Joe is one of my football coaches and he is also a PE teacher. He is a good guy, like everyone thinks a lot of Joe. He focuses a lot on character development and he has told us he would never take a head coach position because he doesn’t want to take too much time away from being the head of the PE department. After school, my buddies and I will have football practice, and I know there is an open gym in the weight room for anyone who wants to lift. There are not many people that go, but there is a chance for them to lift if they want. I have my own car, so when practice is over I drop a few friends off on my way home.

## 4. Discussion

As mentioned earlier, the results of this study, presented as narrative vignettes, are not intended to be generalizable. However, they do provide data-based stories that highlight how young people experience and perceive physical activity opportunities in their schools. Contextually, the three schools that were considered for this study have similarities and differences. For example, they are similar in that they are all secondary schools, and they all engaged with a national physical activity initiative/program. However, they varied greatly when it came to sport facilities, school access (one being a fee-based private school), and opportunities for physical activity at school. Extracurricular activities, especially traditional sport-based opportunities, are much more common in the United States [9] and, to a lesser extent but still present, in Ireland [10]. However, in Finland, such extracurricular activities are generally deemed to be “optional” [11], with the majority of sport participation seeming to happen outside of school in a club environment. This was evident in the results of this study, with students in the United States and Ireland frequently referring to accessing sport and physical activity at school, while the students in Finland only mentioned physical education and time to move during breaks.

Schools are an ideal venue for physical activity participation, and with adequate equipment and sport facilities, schools should provide students with structured and non-structured opportunities for physical activity participation [12]. As illustrated in two of the vignettes, girls in this study generally reported having less opportunity for physical activity at school (except in Ireland), and also less overall participation. These are not novel findings, as they align with historical and current research on girls’ participation in physical activity, especially during secondary school [10]. It should also be noted that girls in this study were more likely to choose physical activities such as walking, which supports previous suggestions that noncompetitive activities have the potential to promote girls’ physical activity [13]. As reported in other studies [14], in addition to walking, some female students in this study (Finland) liked to use the swing-set with their friends. Therefore, secondary schools, which often do not have playground equipment, should consider installing equipment such as swings, especially when considering ways to improve female students’ physical activity levels.

Across all schools, there were some common limitations or barriers to participating in physical activity, including social pressures, school uniforms, access to technology, and student age. Peer pressure, especially for girls, is a barrier that influences students being active in school [14]. This can manifest itself in many ways, including it not appearing “cool” to be active, activity spaces being dominated by competitive play, and students not perceiving themselves to be skilled enough to participate. Pawlowski and colleagues’ [14] study considered the perceptions of children who were approximately 10 years old; therefore, it can be assumed that this peer pressure only increases as children grow older. Similar to findings from other studies [15,16], school uniforms may limit female students’ (specifically in Ireland) physical activity levels. In other words, students who wear more physical activity-friendly uniforms (or clothing), rather than the long skirts required by the studied school in Ireland or jeans worn by many students in the United States, are more likely to be active during breaks [16]. One strategy is to allow students to wear activity-friendly uniforms instead of their traditional uniforms in school.

Another limiting factor was the very evident use of cell phones and other electronics, especially in Finland and the United States. Students who frequently use their cell phones are more likely to forgo opportunities for physical activity in lieu of using their phone [14,17]. To improve students’ physical activity levels, administrators could implement cell phone use policies to limit students’ screen time and media use at secondary schools [18]. However, in this digital age, it might be worth considering innovative ways that technologies, such as mobile phones, could be used to leverage physical activity opportunities rather than simply taking them away. Lastly, most of the students in this study indicated that they were more active in elementary school than they were currently at secondary school. Similar to findings from other studies, students’ physical activity levels decline with increasing age in both females and males [10,12]. However, school culture, environment, and relationships with teachers and peers play an important role in building a positive school community where young people may be more likely to choose to participate in physical activity [12]. It was clear that students in this study were encouraged by their teachers and coaches and that they were influenced by the behaviors of their peers. Therefore, when considering the CSPAP conceptual framework [3], it is important to explore ways to effectively create an active school culture that could positively impact daily participation in physical activity at school.

Additionally, the students and other stakeholders in this study frequently referred to various school-based physical activity opportunities, many of which are evidenced in the narrative vignettes. This supports the concept of a multi-component approach to physical activity promotion, such as the CSPAP model in the United States [3], and a more general whole-school approach [1], which is promoted internationally. While the results of this study provide some information about what students enjoyed about school-based physical activity opportunities, it is also important to examine specific outcomes of such programs. This is especially important to know given that there is evidence that positive health and academic outcomes can result from multi-component whole-school approaches to physical activity promotion [4].

Given the independent characteristics of high school students, schools should consider involving students in decision making related to physical activity opportunities and encourage them to take the lead in the development of physical activity programming and facilities [19]. In this study, this was effective in Ireland, where the transition year students could take an “Active School Flag” class that allowed them to develop physical activity opportunities at their school, and in Finland, where students were consulted in the building of the playground. Involving students in the development of whole-school initiatives in these ways is especially important because there is supporting evidence that suggests that using a co-creational approach, particularly amongst female students, can increase their engagement in physical activity [20].

### Limitations

The results of this study are not intended to be generalized, but they provide a glimpse into the experiences of young people from three different schools in three different countries. Although we collected a wealth of data, these data were only collected over three days (in each site) and it is possible that additional opportunities for physical activity were not observed that may have taken place on Monday or Friday or at different times of the year. Furthermore, stakeholders’ perceptions may have been biased, given that they knew the researcher was there to learn about physical activity opportunities at the schools. Lastly, the field notes and photographs were taken by one person, which contributed to the consistency across sites but could pose a risk of bias.

## 5. Conclusions

Despite there being various opportunities for young people to be active at the secondary schools examined in this study, students believed that there were fewer activities than they had access to in primary/elementary school, and they did not always feel as though the existing opportunities were accessible to them for a variety of reasons (e.g., they were too old, they perceived the activity as non-inclusive, etc.). Given that we know that physical activity levels of children and adolescents decline as they age, it is important to suggest ways in which we can make secondary schools more active places. Whole-school approaches [2], such as the ones employed in the schools studied here, may provide an appropriate framework for creating a more active school culture, and such approaches should be more widely acknowledged within secondary school contexts.

Using narrative vignettes as a methodology allowed us to move beyond simply reporting themes; rather, we attempted to give the reader a more complete story of how young people experience physical activity. While it is important to explore the outcomes of school-based physical activity (e.g., physical activity levels, academic outcomes, etc.), more research should focus on the process of physical promotion and how students engage with physical activity opportunities. Given that students are the intended consumers of school-based physical activity, research in this area should continue to learn more about the experiences of young people with respect to physical activity at school. While this study considered a variety of data from various sources, specific attention was paid to ensuring that the voices of the students who participated in this study were highlighted. Future research should extend this work and continue to explore school-based physical activity opportunities within and across international contexts. Lastly, the results of this study highlight that there are more similarities than differences when it comes to young people’s experiences being active at school, regardless of their country or school setting, and it is likely that we can learn a great deal more from each other, as well as from young people themselves on this topic.

## Figures and Tables

**Table 1:** School information by country.

Country	School Characteristics
Finland	-Lower secondary school (grades 7–9) -Students aged ~13–16 -580 students -Public school -Central Finland
Ireland	-Post-primary school (grades 1st–6th year) -Students aged ~13–19 -600 students -Fee-charging school -Located in West Ireland
United States	-High school (grades 9–12) -Students aged ~14–18 -2094 students -Public school -Located in an upper middle-class neighborhood in a large metropolitan city in the Southwest United States

## Data Availability

The data presented in this study are available on request from the corresponding author. The data are not publicly available to protect participants’ privacy.
